# Supplementary material for: Ocular Surface Characteristics in Pugs with Pigmentary Keratitis in the Canary Islands, Spain
Source: Animals (Basel). 2024 Feb 9;14(4):580. doi: 10.3390/ani14040580 (PMC10885891; doi:10.3390/ani14040580)
Supplement: Supplementary file 1 [file animals-14-00580-s001.zip › animals-2754949-supplementary.pdf]

9. ANEXOS

Anexo I

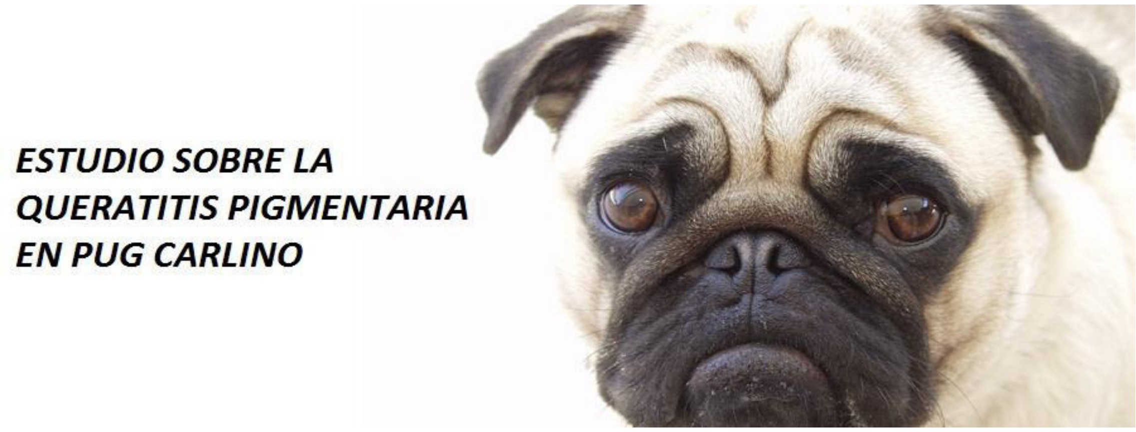

Estimad@ compañer@,

Queremos informarte de la realización de una Tesis doctoral, sobre una alteración ocular muy frecuente en los perros de raza Pug, “la queratitis pigmentaria”.

El objetivo es estudiar la prevalencia, factores predisponentes y signos clínicos de la queratitis pigmentaria en perros de raza Pug, y comparar dos tratamientos inmunosupresores tópicos.

Se les realizará una **exploración oftalmológica gratuita** a todos los Pugs que deseen participar. Si finalmente presentan la alteración podrán ser tratados medicamente **sin coste,** con uno de los dos medicamentos. El estudio durará aproximadamente 6 meses.

La queratitis pigmentaria es una alteración de la córnea en la cual se acumula pigmento, sus causas son multifactoriales entre ellas:

1-La exposición corneal crónica.
2-Las alteraciones de la película lagrimal (queratoconjuntivitis seca).
3-La sensibilidad corneal disminuida.
4-Irritaciones por fricción (entropion, distiquias, triquiasis…).

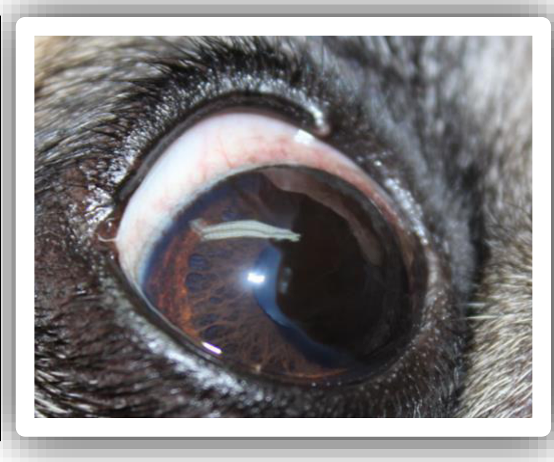

Si tienes algún paciente que pueda beneficiarse pide cita en: diioftalmoanimal@gmail.com
Teléfono: *********

Muchas gracias por la colaboración.
Atentamente,
Diana Sarmiento Quintana, COL. 1032.
Inmaculada Morales Fariña, COL. 85.

Anexo II

Queratitis pigmentaria (PK) en raza Pug   Fecha: /   /

RESEÑA:

Nombre		
Numero de historial /Identificación		
Propietario/ teléfono		/
Edad:	0-5 años	
	5-10 años	
Fecha de nacimiento:	10-15 años	

Sexo	Macho:	
	Hembra:	
Castrado	Sí :	
	No:	
Capa	Plata:	
	Albaricoque:	
*Foto del animal completo	Leonado:	
	Negro :	

ANTENCEDENTES CLINICOS:

OCULARES	Enfermedades anteriores	**Ojo derecho**	**Sí**	**¿Cuál?**
			**No**	
		**Ojo izquierdo**	**Sí**	**¿Cuál?**
			**No**	
	Tratamiento anterior	**Sí**<br>Médicos ☐<br>Quirúrgico ☐	**¿Cuál?**<br>**¿Cuándo?**	
		**No**		

SISTEMICOS	**Sí**	**¿Cuál?**
	**No**	

CÓRNEA: Pigmento que ocupa la córnea

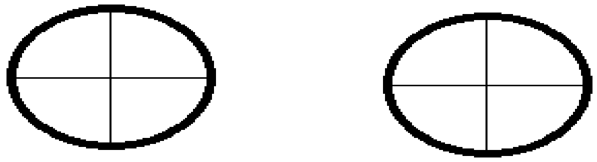

EXPLORACIÓN DEL SEGMENTO ANTERIOR Y ANEJOS:

<table>
<tr><td></td><td colspan="2"></td><td colspan="2">OJO DERECHO</td><td colspan="2">OJO IZQUIERDO</td></tr>
<tr><td rowspan="5">% DE PIGMENTO
QUE OCUPA LA CÓRNEA
*foto de los ojos</td><td colspan="2">Ausencia de PK</td><td colspan="2"></td><td colspan="2"></td></tr>
<tr><td colspan="2">Muy leve</td><td colspan="2">_____ %</td><td colspan="2">_____ %</td></tr>
<tr><td colspan="2">Leve < 20%</td><td colspan="2">_____ %</td><td colspan="2">_____ %</td></tr>
<tr><td colspan="2">Moderada 20-50 %</td><td colspan="2">_____%</td><td colspan="2">_____%</td></tr>
<tr><td colspan="2">Severa > 50 %</td><td colspan="2">_____%</td><td colspan="2">_____%</td></tr>
<tr><td>VASCULARIZACIÓN</td><td colspan="2">SI/NO</td><td colspan="2"></td><td colspan="2"></td></tr>
<tr><td rowspan="8">ANOMALIAS PALPEBRALES

Sí/No</td><td colspan="2">Euribléfaron</td><td colspan="2"></td><td colspan="2"></td></tr>
<tr><td colspan="2">Triquiasis del
pliegue nasal</td><td colspan="2"></td><td colspan="2"></td></tr>
<tr><td colspan="2">Triquiasis de la carúncula</td><td colspan="2"></td><td colspan="2"></td></tr>
<tr><td colspan="2" rowspan="2">Distiquiasis/ distriquiasis
(Poner cuantas)</td><td colspan="2">Superior:</td><td colspan="2">Superior:</td></tr>
<tr><td colspan="2">Inferior:</td><td colspan="2">Inferior:</td></tr>
<tr><td rowspan="2">Entropión
nasal</td><td>Superior</td><td colspan="2"></td><td colspan="2"></td></tr>
<tr><td>Inferior</td><td colspan="2"></td><td colspan="2"></td></tr>
<tr><td colspan="2">Otras:</td><td colspan="2"></td><td colspan="2"></td></tr>
<tr><td rowspan="2">LÁGRIMA</td><td colspan="2" rowspan="2">Test de Schirmer (STT)</td><td colspan="2">mm/min</td><td colspan="2">mm/min</td></tr>
<tr><td>0-5 mm/min</td><td></td><td>0-5 mm/min</td><td></td></tr>
</table>

	CUATITATIVA		5-10 mm/min		5-10 mm/min	
			10-15 mm/min		10-15 mm/min	
			15-20 mm/min		15-20 mm/min	
			20-25 mm/min		20-25 mm/min	
	Test de Ferning (TFT)					
	Break-up time (BUT)		Segundos		Segundos	
			Test fluoresceína:		Test fluoresceína:	
	CUALITATIVA		0-5 s		0-5 s	
			5-10 s		5-10 s	
			10-15 s		10-15 s	
			15-20 s		15-20 s	
SENSIBILIDAD CORNEAL	Fina	Negativa				
		Disminuida				
		Positiva				
	Gruesa	Negativa				
		Disminuida				
		Positiva				
PAQUIMETRÍA ULTRASÓNICA	Zona medial					
	Zona lateral					

		OJO DERECHO	OJO IZQUIERDO
IRIS :	ATROFIA/ HIPOPLASIA		
	PPMs: Poner tipo		
OTRAS:			

Anexo III

UNIVERSIDAD DE LAS PALMAS DE GRAN CANARIA

FACULTAD DE VETERINARIA

HOJA DE CONSENTIMIENTO PARA PARTICIPACIÓN EN

ESTUDIO DE INVESTIGACIÓN

Título: Estudio sobre la queratitis pigmentaria en perros de raza Pug. Comparación de dos tratamientos inmunosupresores tópicos.

Investigador: Diana Sarmiento Quintana

Tutor: Inmaculada Morales Fariña

Lugar: Hospital Clínico Veterinario de la ULPGC

I- INTRODUCCIÓN

Usted ha sido invitado a participar en un estudio clínico. Antes de decidir la participación de su mascota en el estudio por favor lea este consentimiento cuidadosamente. Haga todas las preguntas que usted tenga, para asegurarse de que entienda los procedimientos del estudio.

II- PROPÓSITO DEL ESTUDIO:

La queratitis pigmentaria es una enfermedad ocular muy frecuente en perros de raza Pug, donde el pigmento invade la córnea progresivamente pudiendo provocar pérdida de visión.

El propósito de este estudio es comparar la eficacia de dos tratamientos tópicos, su mascota será tratada con uno de los tratamientos.

III- PARTICIPANTES DEL ESTUDIO:

El estudio es completamente voluntario. Participan aproximadamente 30 perros de raza Pug.

IV- PROCEDIMIENTOS:

El propietario debe poner a su mascota el tratamiento indicado durante un mínimo de 6 meses, tres veces al día.

En este periodo se harán revisiones periódicas, para monitorizar la retirada del pigmento de la córnea, el espesor de la misma y la cantidad y calidad de la lágrima.

V-RIESGOS O INCOMODIDADES:

No hay riesgos especiales que conozcamos en la utilización de la mediación tópica las propias de cualquier medicamento. El propietario entiende los riesgos que puedan ocurrir.

VI- BENEFICIOS

Se espera que el tratamiento tópico frene el avance del pigmento, mejore los signos de inflamación y la cantidad y calidad de la lágrima.

VII- COSTOS

El tratamiento tópico para la queratitis pigmentaria será gratuito, no obstante, si el animal tiene una infección previa será el propietario quien abone la medicación necesaria.

X- PRIVACIDAD Y CONFIDENCIALIDAD

El propietario presta además libremente su conformidad para que se recoja, almacene y analice la información, sobre la salud de su mascota para el propósito de este estudio, sin que revele su identidad personal.

XIV- PREGUNTAS

Si tiene alguna pregunta sobre este estudio o sobre su participación en el mismo, usted puede contactar a: *Diana:* *********

XV- CONSENTIMIENTO:

He leído la información de esta hoja de consentimiento, o se me ha leído de manera adecuada. Todas mis preguntas sobre el estudio y mi participación han sido atendidas.

Yo autorizo el uso y la divulgación de la información sobre la salud de mi mascota. Presto libremente mi conformidad para participar en el estudio.

Al firmar esta hoja de consentimiento, no se ha renunciado a ninguno de los derechos legales.

Don/Doña____ que afirma ser el dueño de la mascota ___________ con microchip __________________________

____

DNI: ____________________

Firma del Participante Fecha

Anexo IV

INSTRUCCIONES PARA EL PROPIETARIO:

Tratamiento:

-Tobrex colirio: 1-1-1 en ambos ojos 7 días y retirar

-Tacrolimus o sirolimus: 1-1-1 en ambos ojos INDEFINIDO. TENER ESTE PRODUCTO EN LA NEVERA Y PROTEGIDO DE LA LUZ

NO utilice ningún otro producto que no esté incluido en este estudio (Por ejemplo: lagrimas artificiales u otros colirios …) sin hablar previamente con Diana Sarmiento Quintana, teléfono: *********

Si se observan legañas SÍ se puede limpiar previamente con suero fisiológico.

Aplíquese el colirio de la siguiente manera:

1. Lávese las manos antes de abrir el envase.
2. Agite el envase
3. Incline la cabeza del perro hacia atrás y que mire hacia arriba.
4. Tire suavemente hacia abajo del párpado inferior, hasta que haga un pequeño hueco.
5. Apriete el envase invertido para que caiga una gota en cada ojo. Para evitar la contaminación no permita que la punta del gotero toque los pelos del animal o cualquier otra superficie. Tape el envase y vuelva a introducirlo en el cilindro de plástico para que el producto este protegido de la luz.
6. La primera semana junto al Tobrex colirio. Esperar cinco minutos entre los dos productos.

Si cree que una gota cayó fuera del ojo repita la operación.

Control telefónico con cualquier incidencia.

Anexo V

REVISIONES Fecha: / /

RESEÑA:

Nombre	
Identificación	
Propietario/ teléfono	

PRODUCTO: **OD** **OS**

LÁGRIMA	Test de Schirmer (STT)	mm/min		mm/min	
	CUATITATIVA	0-5 mm/min		0-5 mm/min	
		5-10 mm/min		5-10 mm/min	
		10-15 mm/min		10-15 mm/min	
		15-20 mm/min		15-20 mm/min	
		20-25 mm/min		20-25 mm/min	
	Test de Ferning (TFT)				
	Break-up time (BUT)	Segundos		Segundos	
		Test fluoresceína:		Test fluoresceína:	
	CUALITATIVA	0-5 s		0-5 s	
		5-10 s		5-10 s	
		10-15 s		10-15 s	
		15-20 s		15-20 s	
PAQUIMETRIA	Zona medial				
En el lateral	Zona sin pigmento				

OTRAS OBSERVACIONES

CÓRNEA: PK

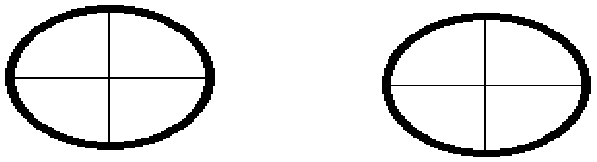

SIGNOS CLÍNICOS E INDICADORES DE EFECTIVIDAD

	Ojo derecho (OD)					Ojo izquierdo (OS)				
	Ausencia (1)	Ligera (2)	Moderada (3)	Avanzada (4)	Grave (5)	Ausencia (1)	Ligera (2)	Moderada (3)	Avanzada (4)	Grave (5)
**SIGNOS CLINICOS**										
BLEFAROESPASMO										
HIPEREMIA										
PRURITO										
EDEMA										
INFILTRADO										
VASOS										
PIGMENTO EN ESCLERA										
DESCARGA	Ausencia	Serosa	Mucosa	Purulenta		Ausencia	Serosa	Mucosa	Purulenta	
**INDICADORES EFECTIVIDAD**										
LINEA TRANSPARENTE	Ausencia (1)	Leve (2)	Moderada (3)	Intensa (4)		Ausencia (1)	Leve (2)	Moderada (3)	Intensa (4)	
PIGMENTO ACLARADO	Sin aclarado	Aclarado leve	Aclarado moderado	Aclarado intenso		Sin aclarado	Aclarado leve	Aclarado moderado	Aclarado intenso	
ÚLCERAS	Sí		No			Sí		No		

RECUPERACIÓN DE TRANSPARENCIA A LOS 6 MESES

FECHA: /   /

Nombre	
Identificación	
Propietario	
Producto	

	Ojo derecho (OD)				Ojo izquierdo (OS)			
	No	Leve	Moderada	Intensa	No	Leve	Moderada	Intensa
**Recuperación de Trasparencia**								
